# Evaluating the Effectiveness of Robotic Process Automation for Cancer Registry Data Abstraction in a Production EHR Environment

**DOI:** 10.3390/jcm15072657

**Published:** 2026-03-31

**Authors:** Se Young Jung, Jong Soo Han, Kihyuk Lee, Ho-Young Lee

**Affiliations:** 1 Department of Family Medicine, Seoul National University Bundang Hospital, Seongnam 13620, Republic of Korea; syjung@snubh.org (S.Y.J.); jeremy.han@snubh.org (J.S.H.); 2 Department of Family Medicine, College of Medicine, Seoul National University, Seoul 03080, Republic of Korea; 3 Office of Hospital Information, Seoul National University Bundang Hospital, Seongnam 13605, Republic of Korea; 4 Health Promotion Center, Seoul National University Bundang Hospital, Seongnam 13620, Republic of Korea; 5 Office of eHealth Research and Business, Seoul National University Bundang Hospital, Seongnam 13605, Republic of Korea; 6 Department of Nuclear Medicine, Seoul National University Bundang Hospital, Seongnam 13620, Republic of Korea; 7 Department of Nuclear Medicine, College of Medicine, Seoul National University, Seoul 03080, Republic of Korea

**Keywords:** robotic process automation (RPA), cancer registry, electronic health records (EHR), workflow efficiency, qualitative research

## Abstract

**Background/Objectives:** Robotic Process Automation (RPA) offers a potential solution for reducing the manual burden of clinical data abstraction, yet empirical evidence of its effectiveness in real-world electronic health record (EHR)-integrated cancer registries remains limited. This study aimed to evaluate the post-implementation effectiveness of RPA for cancer registry data abstraction in a tertiary hospital and to explore multidisciplinary stakeholder perceptions regarding its deployment. **Methods:** We implemented RPA for gastric and breast cancer registries within a production EHR system. Quantitative effectiveness was evaluated by comparing per-patient data extraction time using descriptive statistics. To ensure data integrity, all RPA-extracted outputs were entirely verified manually by researchers against source records. Qualitatively, semi-structured interviews were conducted with 14 participants and analyzed via thematic analysis based on the Promoting Action on Research Implementation in Health Services (PARiHS) framework (Evidence, Context, and Facilitation). **Results:** RPA was applied to 70 gastric cancer variables and 83 breast cancer variables. For the gastric cancer registry, the mean abstraction time per patient decreased by 74% (19.5 ± 3.0 to 5.1 ± 1.8 min). For the breast cancer registry, time decreased by 30% (25.4 ± 6.9 to 17.8 ± 5.5 min). Based on 2024 surgical volumes, this translates to an estimated saving of over 260 h of manual labor per year. Qualitative findings revealed that while participants recognized RPA as ideal for repetitive tasks, successful implementation was contingent on clinician cooperation and continuous output monitoring. **Conclusions:** RPA implementation significantly improved data abstraction efficiency in a real-world clinical research workflow. The disparity in time savings highlights that efficiency gains are contingent upon registry complexity. While formal quantitative assessments of data accuracy were not performed, RPA is a readily deployable tool for enhancing clinical data workflows when aligned with organizational readiness and robust monitoring.

## 1. Introduction

Electronic health records (EHRs) are digital collections of patient health information, including demographics, diagnoses, and medical histories, among other data [1,2]. EHR systems have become widely adopted across healthcare institutions in South Korea and the United States, reflecting their central role in contemporary clinical practice and healthcare quality improvement [3,4,5]. The widespread use of EHRs has enabled more systematic documentation of clinical care and has expanded opportunities for secondary use of clinical data in research, quality assessment, and registry development.

EHRs facilitate easier access to clinical information for secondary purposes compared to traditional paper charts. However, the data often require preprocessing to ensure accuracy due to their disparate and incomplete nature [6]. Although clinical data warehouses (CDWs) were introduced to support secondary use of EHR data, substantial manual preprocessing remains necessary, particularly for registry-oriented data abstraction, because a large proportion of clinically relevant information is embedded in unstructured text [7,8,9].

Despite these advances, extracting structured clinical and registry variables from EHRs remains highly labor-intensive and time-consuming, frequently demanding substantial manual effort from trained personnel. This challenge is especially pronounced in disease registries, such as cancer registries, where abstractors must repeatedly identify, verify, and transcribe predefined variables following strict and consistent rules [10,11]. These characteristics position registry data abstraction as a persistent operational bottleneck, limiting the scalability and timeliness of registry-based clinical research.

Robotic process automation (RPA) has recently emerged as a promising approach to automate repetitive, rule-based tasks in healthcare by replicating predefined human interactions with information systems. Internationally, its adoption has expanded in administrative domains such as billing, scheduling, and claims processing [12]. However, the application of RPA to clinical registry data abstraction, where accuracy, consistency, and sustained workload reduction are critical, remains limited and underexplored [13,14,15].

While a previous study demonstrated the feasibility of applying RPA to hospital business process monitoring [16], this study extends its application to structured clinical research data workflows. Although earlier RPA studies emphasized administrative efficiency, empirical evidence quantifying its effectiveness in registry-based clinical abstraction, where standardized rules represent a suitable target for automation, remains scarce [12,17,18,19]. Because clinical registries rely on repetitive, rule-based processing of predefined variables, they represent a timely and underexplored target for evaluating the post-implementation effectiveness of RPA within real-world EHR environments.

Accordingly, this study aims to evaluate the post-implementation effectiveness of RPA for cancer registry data abstraction in a South Korean tertiary hospital, with a focus on quantitative improvements in data extraction efficiency and qualitative insights into its implications for clinical research data workflows, specifically in registry-oriented tasks rather than general process monitoring.

## 2. Materials and Methods

### 2.1. Research Design and Environment

This study was conducted in a South Korean tertiary hospital. Because the hospital has experience in medical information and communication technology (ICT) development projects, members of the medical informatics team are actively engaged in introducing new medical ICT [7,20,21]. The medical information team in this institution is an administrative department responsible for managing and archiving patient medical records, and it operates in close collaboration with the Information Technology (IT) department to implement and manage digital health systems. We used a mixed-methods approach consisting of both quantitative and qualitative components [16].

For the quantitative component, the efficacy of RPA was evaluated by comparing the time required to extract predefined registry information using either manual methods or RPA. Because both the manual approach and RPA used the same predefined data format, our primary performance metric was the time required for data extraction. The RPA utilized in this study was strictly limited to rule-based extraction and did not involve any natural language processing or inference. All extracted data were reviewed by human personnel to ensure accuracy.

### 2.2. Overview of the RPA Architecture

A task force team (TFT) performed an iterative process to implement the system-monitoring RPA bot. The role of the TFT was to design an RPA bot by analyzing hospital operating system workflows and subsequently evaluating the effectiveness of the developed RPA. The environment for the RPA bot was configured to mirror the manual data preparation workflow to ensure direct comparability between manual and automated extraction.

**Figure 1:**
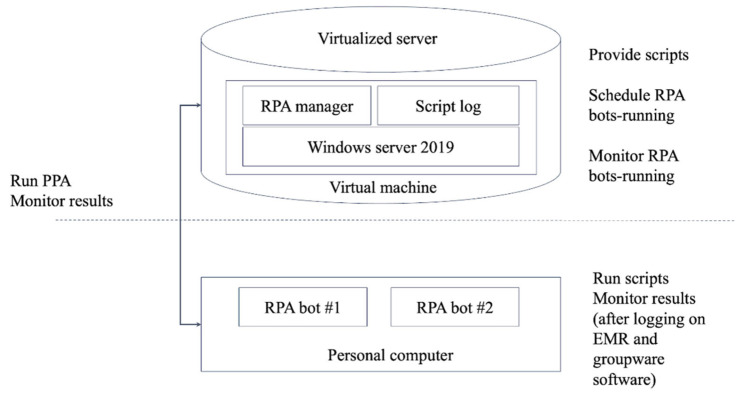
Overall architecture of RPA.

The RPA system consisted of a virtualized server environment and dedicated RPA execution computers, as illustrated in Figure 1. The RPA bot was developed using the AutomateOne platform (version 4.22.12, Gridone Inc., Seoul, Republic of Korea). The virtual server hosted the RPA manager and script log modules, while the RPA bots were deployed on local computers that logged into the hospital’s electronic medical record (EMR) and groupware systems to run scripts under the same operational conditions as manual abstraction.

The RPA project in our study began in August 2020 and extended until October 2020, totaling three months dedicated to coding, testing, and finalizing the system. This period was crucial for ensuring that the RPA system was tailored to the specific needs of the hospital and that it would operate efficiently and reliably once launched. Following this foundational phase, the RPA has been in stable operation, seamlessly integrating into the hospital’s workflow.

### 2.3. Interview Framework and Qualitative Approach

In addition to the quantitative evaluation, in-depth interviews were conducted to explore factors related to successful RPA implementation in research data abstraction. A medical information team interviewed nursing, administrative, and technical personnel to gather perspectives from diverse stakeholders involved in RPA operations.

The interview questionnaire was developed according to the Promoting Action on Research Implementation in Health Services (PARiHS) framework [22]. The PARiHS framework conceptualizes successful implementation (SI) as a function of evidence (E), context (C), and facilitation (F): SI = f(E, C, F) [22]. Based on this model, a semi-structured interview guide was created (Supplementary Table S1). Participants were briefly introduced to the fundamental concepts of RPA prior to the interviews to ensure adequate background knowledge. Qualitative reporting adhered to the consolidated criteria for reporting qualitative research (COREQ) guideline [23] (Supplementary Table S2).

### 2.4. Participant Recruitment

The participants were recruited using both purposeful and snowball sampling. The purposeful sampling method, which is a nonprobable sampling approach in which the researcher selects a sample based on their judgment [24], was used to select the initial interviewees. Snowball sampling, another nonprobable method where enrolled participants help recruit future participants, was subsequently employed. This method is valuable when identifying eligible members of the target population is challenging [25]. Two personnel in administrative positions and one medical information team developer were selected as early participants, as they had been involved in the RPA implementation process from the beginning. Following purposive sampling, snowball sampling was used to identify and interview additional individuals recommended by the preceding participants.

### 2.5. Data Collection (Quantitative and Qualitative)

For the qualitative component, the audio-recorded interviews were transcribed verbatim by a professional transcription service and independently reviewed by two researchers to verify accuracy. Each researcher coded key statements corresponding to the domains of the PARiHS framework, and discrepancies were resolved through discussion. Coding was independently conducted by the two researchers, and differences were addressed through discussion to ensure consistency. To mitigate social desirability bias, interviews were conducted by an independent researcher who was not directly involved in the RPA development team. Participants were assured of anonymity and confidentiality, and it was emphasized that their feedback would be used solely for research purposes to improve system implementation. Data triangulation was achieved by comparing interview themes with quantitative performance metrics to ensure the trustworthiness of the findings.

For the quantitative component, we examined the data preparation workflow of the cancer registry. In routine practice, one trained researcher in each clinical department manually reviews demographic information, preoperative assessments, surgical information, pathology results, and postoperative treatment history in the EHR system and enters these into a predefined clinical research form (CRF). Binary variables are recorded as yes or no, and predefined continuous variables are entered as numeric values. To minimize secular bias, both evaluations were conducted using the same EHR version and identical registry forms, performed by the same group of trained research personnel.

**Figure 2:**
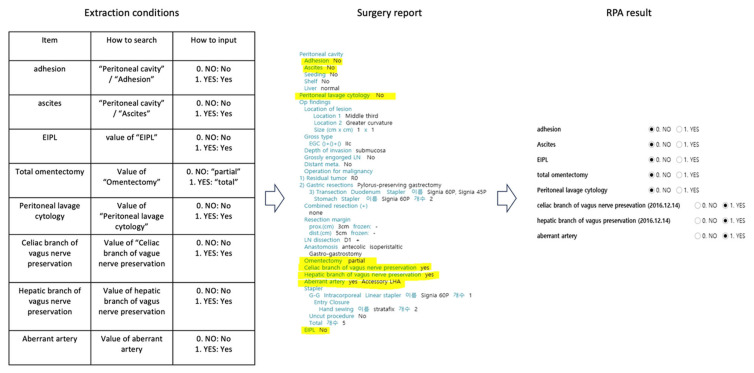
Example of extraction by RPA.

The quantitative evaluation included a total of 31 cases for the gastric cancer registry and 24 cases for the breast cancer registry, measured during a stable operation period from 19 October 2020 to 9 November 2020. The automation boundary was defined by data structure: RPA handled binary and numeric variables from structured surgery and pathology reports (Figure 2), while complex free-text variables remained manual. Observed failure modes, such as EHR format drift or system latency, were mitigated through the manual verification process (α). As detailed in Supplementary Table S3, the gastric cancer registry focused on 70 objective variables to ensure high abstraction reliability.

The selected registries represent the highest volume of cases in our institution, providing a sufficient dataset for evaluation. Furthermore, they contain a diverse mix of structured data and unstructured text, which is ideal for testing RPA versatility in different abstraction scenarios. The RPA application was strategically focused on quantitative and objective items (70/168 variables for gastric cancer; 83/121 for breast cancer) to minimize human error. Following a predefined protocol, complex variables requiring professional clinical judgment or those with unpredictable text patterns (e.g., specific postoperative complications) were excluded from the automation scope and managed manually. To ensure 100% data integrity, all RPA-extracted outputs underwent full manual verification against original EHR source records. The reported average working times (AWT) after RPA include this human review, error correction, and the manual entry of non-automated variables (α). All cases in both eras followed the same inclusion criteria (standardized registry requirements), with no significant differences in case mix or data complexity. Extraction time was measured using a consistent timing procedure in the same computing environment for all cases to ensure comparability.

An illustrative screenshot demonstrates the RPA’s ability to extract predefined registry variables, particularly binary items, from unstructured surgery reports (Figure 2). The example shows a sample extraction from a surgery report with all personal health information redacted.

Data extraction time was measured after the RPA system had reached stable routine operation and was compared with historical manual extraction performed using the same predefined registry format.

### 2.6. Statistical Analysis

For the quantitative analysis, all descriptive statistics were calculated using IBM SPSS Statistics for Windows, Version 25.0 (IBM Corp., Armonk, NY, USA). Descriptive statistics (mean and range) were used to evaluate the efficiency gains in data extraction time. Due to the distinct nature of the data sources, where the pre-RPA baseline represents an institutional performance benchmark and the post-RPA data consist of prospectively measured individual cases (31 for gastric cancer and 24 for breast cancer), we prioritized a descriptive comparison of values over formal hypothesis testing. This approach was chosen to accurately reflect the practical magnitude of workload reduction in a real-world clinical setting without the potential for statistical overinterpretation. Results are presented as the absolute and percentage reduction in extraction time.

For the qualitative analysis, interview transcripts were reviewed and coded by two researchers. A thematic analysis approach was used to identify key concepts related to organizational factors influencing RPA implementation. Codes were grouped into higher-level themes corresponding to the PARiHS framework domains (evidence, context, and facilitation). Representative quotations were selected to illustrate each theme.

### 2.7. Ethical Approval

This study was approved by the Institutional Review Board of Seoul National University Bundang Hospital (SNUBH) (IRB No. B-2206-760-306). In accordance with IRB requirements, all interview participants were informed of the study’s purpose, voluntary participation, and their right to withdraw at any time without consequence. Written or recorded verbal consent was obtained prior to each interview. All qualitative and quantitative data were de-identified before analysis, and no personal identifiers were included in the final dataset. For the quantitative component, the study utilized retrospective, de-identified data in accordance with institutional data governance policies. All data extraction and timing evaluations were conducted within the hospital’s secure internal network, with no data leaving the institution. Furthermore, the RPA bot operated under dedicated accounts with restricted access, fully monitored by the hospital’s standard auditing and access control systems. The study was conducted in compliance with the ethical principles outlined in the Declaration of Helsinki and institutional data-protection policies.

## 3. Results

### 3.1. Study Population Characteristics

In-depth interviews were conducted with a total of 14 participants involved in the development, operation, and coordination of the hospital information system. The mean age of the participants was 41.4 years, and 64% were male. With the exception of one participant, all had more than five years of professional experience, and 64% reported over ten years of work experience.

Participants represented multidisciplinary roles within the medical information team, including nursing personnel (14%), administrative staff (21%), and developers (65%). Nursing personnel primarily coordinated and communicated clinical requirements to the development team, while administrative staff facilitated collaboration between clinical and technical teams, and developers were responsible for the development and maintenance of the hospital information system. Detailed demographic and professional characteristics of the study participants are summarized in Table 1.

### 3.2. Qualitative Findings Based on the PARiHS Framework

The qualitative interview findings were analyzed using the PARiHS framework, which comprises three core components: evidence, context, and facilitation. Across all participant groups, common themes were identified within each PARiHS component, reflecting shared perceptions of RPA and its implementation environment (Table 2).

Under the Evidence domain, participants consistently reported prior exposure to or background knowledge of RPA, indicating a common understanding of RPA as a technology suited for simple and repetitive tasks. This perception was observed across nursing, administrative, and developer roles.

Within the Context domain, participants described the hospital environment as supportive of RPA adoption, particularly in relation to reducing repetitive data entry and improving operational efficiency. However, differences by role were observed, with nursing and administrative staff emphasizing the need for clinician cooperation in identifying and extracting relevant clinical information, while developers highlighted the technical stability of the hospital information system as an important contextual factor.

Regarding Facilitation, participants commonly identified the importance of monitoring RPA-generated outputs and managing potential risks associated with system changes. These facilitating factors were consistently mentioned across roles, although their specific concerns varied depending on professional responsibilities.

A summary of qualitative findings categorized by PARiHS components and participant roles is presented in Table 2.

Representative interview excerpts supporting the identified themes across PARiHS domains and job roles are provided in Supplementary Table S4.

### 3.3. Effectiveness of RPA for Cancer Registry Data Abstraction

RPA was applied to a subset of predefined registry variables for the creation of gastric and breast cancer registries. Detailed item-level information is presented for the gastric cancer registry as a representative example (Supplementary Table S3). For the gastric cancer registry, RPA was applied to 70 variables, and the average data extraction time per patient was 19.5 ± 3.0 min before RPA implementation, which was reduced to 5.1 ± 1.8 min after RPA implementation, representing a 74% reduction in processing time (*p* < 0.01). For the breast cancer registry, RPA was applied to 83 variables, and the average data extraction time per patient was 25.4 ± 6.9 min before RPA implementation, which was reduced to 17.8 ± 5.5 min after RPA implementation, corresponding to a 30% reduction in processing time (*p* < 0.01). The extent of automation varied by registry component, including demographic information, preoperative evaluation, operation records, pathological reports, and postoperative data. Details regarding the number of variables subject to RPA and changes in average work time before and after RPA implementation are summarized in Table 3.

## 4. Discussion

This study evaluated the post-implementation effectiveness of robotic process automation (RPA) for cancer registry data abstraction in a tertiary hospital environment and demonstrated both quantitative efficiency gains and qualitative organizational insights. Unlike previous RPA studies that primarily focused on conceptual feasibility or simulated workflows, this study examined operational performance after real-world deployment in a production EHR system. RPA reduced per-patient data abstraction time by 73.8% for the gastric cancer registry (70 variables) and by 29.9% for the breast cancer registry (83 variables). To contextualize these findings in practical terms, we estimated the annual impact based on our institution’s 2024 surgical volume (approximately 600 gastric and 950 breast cancer cases). The RPA implementation could potentially save over 260 h of manual labor annually. This reduction allows research personnel to reallocate their time toward higher-value tasks, such as complex data validation and clinical quality improvement, rather than repetitive data entry.

These results indicate that efficiency gains varied according to registry complexity and documentation characteristics rather than reflecting uniform automation effects. This disparity in efficiency reflects the inherent challenge of extracting information from breast cancer records, which often contain more fragmented and complex narrative data compared to the more standardized gastric cancer templates [26,27]. As clinical documentation for breast cancer frequently involves multifaceted parameters across diverse report types, the RPA bot requires more extensive navigation, thereby limiting the relative time savings [8]. However, it should be noted that this interpretation is post hoc, as a formal quantitative comparison between structured and narrative variable proportions across the two registries was not conducted in this study.

Only a limited number of studies have examined RPA applications in clinical contexts beyond administrative processes. Thainimit et al. reported reduced screening time and improved throughput after applying RPA to a glaucoma screening system [28], but their evaluation focused on system-level outcomes rather than post-implementation workload reduction for structured data abstraction. Jerry et al. demonstrated faster processing of antibiotic susceptibility test results using RPA [29], though the application was limited to a narrowly defined diagnostic task. Kobayashi et al. described conceptual use cases of RPA-enabled robots in elderly care [30], and Sreekrishna et al. explored RPA-based automation for extracting cancer pathology information [15], but both studies emphasized feasibility or pilot implementation without a quantitative assessment of routine data workflow efficiency.

Several studies have suggested that RPA may improve hospital logistics and administrative processes. Liu et al. evaluated RPA for hospital logistics using simulation scenarios [31], while Kim et al. reported a reduced administrative burden after introducing RPA into insurance claim self-inspection workflows in Korea [32]. Ratia et al. explored perceptions of RPA adoption through interviews and highlighted its potential for administrative support [33]. However, these studies largely relied on simulations, interviews, or pre-implementation assessments rather than measured post-deployment effects in real-world EHR-based clinical data workflows.

A recent hospital-based study further demonstrated the feasibility of applying RPA to hospital information systems for business process monitoring using a mixed-methods approach [16]. That study primarily focused on operational monitoring and organizational feasibility within hospital information systems. Building on this prior work, the present study extends the application of RPA from administrative and operational monitoring to structured clinical research data workflows, specifically cancer registry data abstraction, and quantitatively evaluates post-implementation effectiveness at the level of individual registry variables.

In contrast, the present study quantitatively evaluated the effects of RPA after its deployment within disease-specific cancer registry workflows, demonstrating efficiency gains at the level of individual registry variables. Given that clinically relevant information is frequently embedded in free-text narratives [26,27], and clinical documentation is inherently unstructured [8], registry data preparation continues to rely heavily on manual review and abstraction by trained personnel [11]. This reliance on manual processes has been consistently identified as one of the most time- and labor-intensive components of clinical research and quality management workflows [34], further highlighting the operational necessity of automation tools like RPA.

Database-level extraction and schema-based automation approaches have been proposed to address these challenges [7]. However, in real-world operational environments, such approaches often require substantial human involvement for semantic mapping, data standardization, and ongoing integration and maintenance [35,36,37,38,39]. More recently, machine learning and natural language processing-based methods have been suggested as promising alternatives for improving the secondary use of EHR data [3,40]. Despite their potential, these approaches frequently depend on highly structured data environments, institution-specific model development, or additional infrastructure optimization, which can limit their immediate applicability to routine registry workflows [41,42,43]. In this study, RPA was not intended to replace or compete with machine learning-based extraction approaches but was instead adopted to address an operational need for immediate workload reduction in predefined, rule-based registry tasks within existing EHR interfaces.

RPA addresses a unique operational niche by replicating human interactions at the user interface level without altering underlying database structures. In addition to the observed quantitative efficiency gains, our qualitative findings provide contextual evidence for its effectiveness; across stakeholder groups, RPA was consistently perceived as a technology ideally suited for simple, rule-based, and repetitive tasks. Participants emphasized that successful deployment depended on clear task definition, segmentation of workflows into repeatable units, and continuous monitoring of automated outputs. These organizational insights extend prior hospital-based RPA research, which primarily focused on business process monitoring [16], by expanding the application domain to structured clinical research data entry. By demonstrating stable post-implementation performance, this study provides empirical evidence that RPA can move beyond operational monitoring to support clinically meaningful research data workflows when task characteristics, user perceptions, and organizational readiness are appropriately aligned.

Several limitations should be considered. First, this study was conducted at a single tertiary hospital, which may limit the generalizability of the findings to other institutional settings with different information systems or registry workflows. Second, although all RPA-extracted data underwent manual verification by researchers, data accuracy and completeness metrics (e.g., error rates) were not systematically quantified or analyzed; future studies should incorporate formal validation using standardized accuracy assessments. Third, the qualitative findings were based on interviews with a limited number of participants involved in system development and operation, which may not fully capture the perspectives of all end users. Fourth, this study compared prospectively measured post-RPA data against baseline figures derived from historical institutional logs. The use of historical records for pre-RPA manual extraction times, rather than a prospective controlled comparison, may introduce measurement bias. Future studies with a more rigorous concurrent control group design could provide a more precise assessment of the intervention’s impact. Lastly, some COREQ items, such as participant checking and field notes, were omitted due to logistical constraints. While RPA offers significant efficiency gains, the implementation effort and maintenance burden must be considered. As highlighted in the interviews, RPA systems are sensitive to EHR format changes (format drift), requiring continuous monitoring and periodic updates to ensure sustained performance. Despite these limitations, the study provides practical insight into the conditions under which RPA can be effectively implemented in registry-oriented clinical research workflows.

## 5. Conclusions

In conclusion, the implementation of RPA for cancer registry data abstraction was associated with meaningful reductions in data extraction time and positive organizational perceptions in a real-world hospital setting. By demonstrating post-implementation efficiency gains at the level of individual registry variables within disease-specific workflows, this study shows that RPA can be an effective and readily deployable approach for supporting clinical research data workflows that rely on predefined, repetitive, and rule-based tasks. Future research should evaluate data quality outcomes, cost-effectiveness, and multi-institutional applicability to further clarify the role of RPA in clinical informatics and registry-based research.

## Figures and Tables

**Table 1:** Characteristics of study participants.

Variables	*n* (%)
Mean age	41.4 ± 5.6
Sex	
Male	9 (64)
Female	5 (36)
Job kind	
Medical information team, nursing position	2 (14)
Medical information team, administrative position	3 (21)
Medical information team, developer	9 (65)
Years of work	
Less than 5 years	1 (7)
Between 5 and 10 years	4 (29)
10 years or more	9 (64)

Data are presented as mean ± standard deviation for numerical variables and numbers (percentages) for categorical variables.

**Table 2:** Summary of study participants based on the PARiHS framework.

PARiHS Component	Nursing Position	Administrative Position	Developers
Evidence			
Prior experience and background knowledge	Yes	Yes	Yes
Context			
Environment	RPA replaces repetitive input operations to increase operational efficiency	Need clinicians’ cooperation to extract keywords	RPA replaces repetitive input operations to increase operational efficiency
Attitude	Positive	Positive	Positive, partially negative
Facilitating factors			
Anticipated risks	Need a way to monitor RPA-generated results	Need a way to monitor RPA-generated results	If the hospital information system screen or environment changes frequently, it is difficult to apply RPA
Suggestion			
	Step-by-step RPA improvement with Power Users is needed	Improvement of RPA based on RPA project experience is needed	Establishment of a process for target operations is needed

**Table 3:** Comparison of the time to create a gastric and breast cancer registry before and after the introduction of RPA.

Type	Component	Application of RPA	Variables Automated/Total Variables	AWT Before RPA (min)	AWT After RPA (min)	Reduction (%)
Gastric cancer (N = 31)	Demographic	Partially Yes ^†^	0/12	19.5 ± 3.0	5.1 ± 1.8	73.8%
	Preoperative Evaluation	Yes	19/28			
	Operation Record	Yes	27/34			
	Pathologic Report	Yes	24/25			
	Postoperative	No	0/69			
	Total components		70/168			
Breast cancer (N = 24)	Demographic	Yes	14/28	25.4 ± 6.9	17.8 ± 5.5	29.9
	Preoperative Evaluation	Yes	30/34			
	Operation Record	Yes	5/9			
	Pathologic Report	Yes	19/20			
	Postoperative	Yes	15/30			
	Total components		83/121			

Data are presented as mean ± SD or %. N indicates the number of clinical records evaluated for timing during the post-RPA pilot period. The “Variables automated/Total variables” column represents the ratio of variables with full or partial automation relative to the total number. ^†^ “Yes” indicates full automation from data extraction to entry; “Partially Yes” indicates components for which RPA was applied to navigational workflows (e.g., accessing specific EMR menus) or automatic data transfer, requiring human intervention to initiate the script or manually verify specific fields that rule-based logic could not fully parse; “No” indicates items that were entirely abstracted by hand due to high complexity or a lack of standardized rules. RPA, robotic process automation; AWT, average working time.

## Data Availability

The datasets generated and analyzed during the current study are not publicly available due to strict institutional data-protection policies regarding electronic health records. Access to these data is limited to authorized personnel within the institution to ensure the highest level of patient confidentiality.

## Supplementary Materials

The following supporting information can be downloaded at: https://www.mdpi.com/article/10.3390/jcm15072657/s1, Table S1: Interview questions; Table S2: Consolidated criteria for reporting qualitative studies (COREQ): 32-item checklist; Table S3: Detailed items of gastric cancer registry; Table S4: Results of in-depth interviews.
