# Supplementary material for: Evaluating the Effectiveness of Robotic Process Automation for Cancer Registry Data Abstraction in a Production EHR Environment
Source: J Clin Med. 2026 Mar 31;15(7):2657. doi: 10.3390/jcm15072657 (PMC13073201; doi:10.3390/jcm15072657)
Supplement: Supplementary file 1 [file jcm-15-02657-s001.zip › jcm-4174244-supplementary.pdf]

Supplementary Table S1. Interview questions

Questions about Prior experience and background knowledge on RPA

"Have you ever participated in RPA project?”

“Have you ever heard about RPA?”

Questions about Robotic Process Automation

"What do you think is the most impressive part of the RPA? What do you think sets you apart from traditional business automation?”

"How can RPA contribute to the automation of SNUBH's work? Are there pros and cons in that respect?”

“Do you think SNUBH was ready to implement RPA?

“What’s your attitude toward RPA?

"Which RPA project do you think is the most important?"

"What efforts are needed to operate and maintain the RPA well? Organizational structure? System Maintenance? What did you feel differently from traditional task automation?”

Question about cancer registry recording by RPA.

"What do you think about the task of reconstructing patient information? Do you have any related work or experience?"

"Creating cancer patient information was costly. Why hasn't it been automated?”

"Is the RPA program useful for recording cancer patient information?"

What are the facilitating factors or anticipated risks for the RPA project?

What else do you think we need to implement RPA more successfully than before?

Supplementary Table S2. Consolidated criteria for reporting qualitative studies (COREQ): 32-item checklist.

**No. Item**	**Guide questions/description**	**Reported on Page #**
**Domain 1: Research team and reflexivity**		
Personal Characteristics		
1.Inter viewer/facilitator	Which author/s conducted the interview or focus group?	Methods<br>p.5
2. Credentials	What were the researcher's credentials? E.g. PhD, MD	Methods p.5
3. Occupation	What was their occupation at the time of the study?	Methods p.5
4. Gender	Was the researcher male or female?	N/A
5. Experience and training	What experience or training did the researcher have?	Methods p5
Relationship with participants		
6. Relationship established	Was a relationship established prior to study commencement?	N/A
7. Participant knowledge of the interviewer	What did the participants know about the researcher? e.g. personal goals, reasons for doing the research	N/A

8. Interviewer characteristics	What characteristics were reported about the inter viewer/facilitator? e.g. Bias, assumptions, reasons and interests in the research topic	N/A
**Domain 2: study design**		
Theoretical framework		
9. Methodological orientation and Theory	What methodological orientation was stated to underpin the study? e.g. grounded theory, discourse analysis, ethnography, phenomenology, content analysis	Methods<br>p.5
Participant selection		
10. Sampling	How were participants selected? e.g. purposive, convenience, consecutive, snowball	Methods<br>p.5
11. Method of approach	How were participants approached? e.g. face-to-face, telephone, mail, email	Methods<br>p.5
12. Sample size	How many participants were in the study?	Results

13. Non-participation	How many people refused to participate or dropped out? Reasons?	N/A
Setting		
14. Setting of data collection	Where was the data collected? e.g. home, clinic, workplace	Methods pp.4-5
15. Presence of non-participants	Was anyone else present besides the participants and researchers?	N/A
16. Description of sample	What are the important characteristics of the sample? e.g. demographic data, date	Results p.7
Data collection		
17. Interview guide	Were questions, prompts, guides provided by the authors? Was it pilot tested?	N/A
18. Repeat interviews	Were repeat inter views carried out? If yes, how many?	N/A
19. Audio/visual recording	Did the research use audio or visual recording to collect the data?	Methods pp.5

20. Field notes	Were field notes made during and/or after the inter view or focus group?	N/A
21. Duration	What was the duration of the interviews or focus group?	Methods<br>pp.5
22. Data saturation	Was data saturation discussed?	N/A
23. Transcripts returned	Were transcripts returned to participants for comment and/or correction?	N/A
**Domain 3: analysis and findings**		
Data analysis		
24. Number of data coders	How many data coders coded the data?	Methods<br>pp.5
25. Description of the coding tree	Did authors provide a description of the coding tree?	N/A
26. Derivation of themes	Were themes identified in advance or derived from the data?	Results<br>pp.7-10

27. Software	What software, if applicable, was used to manage the data?	N/A
28. Participant checking	Did participants provide feedback on the findings?	N/A
Reporting		
29. Quotations presented	Were participant quotations presented to illustrate the themes/findings? Was each quotation identified? e.g. participant number	Results pp.7-10
30. Data and findings consistent	Was there consistency between the data presented and the findings?	Results pp.7-10
31. Clarity of major themes	Were major themes clearly presented in the findings?	Results pp.7-10
32. Clarity of minor themes	Is there a description of diverse cases or discussion of minor themes?	Results pp.7-10

Supplementary Table S3. Detailed items of gastric cancer registry

Domain	Items
General information	Past Medical History Details
	Preoperative endoscopic mucosal resection or submucosal dissection
	History of malignancy of other organs
	Malignant organs
	Primary cancers in other organs details
	History of abdominal operations
	Previous abdominal surgery details
	History of gastric operations
	Previous gastric surgery details
	Familiar history or gastric cancer
	Smoking history: Pack/years
	Preop. Chemotherapy
	Chief complaint
	Preop. CT T stage
	Preop. CT N stage
	Preop. CT M stage
	Preop. EUS T stage
	Preop. EUS N stage
Operation Record	Intent of operations
	Radicality
	Open, Laparoscopic, Robot
	Reason for open conversion
	Estimated blood loss (mL)
	Intraoperative Transfusion

	Transfusion information detail
	Type of operations
	Type of operations detail
	Reconstruction
	Extra,Intra-corporeal reconstruction of main anastomosis
	Transgastric wedge resection
	Single incisional laparoscopic surgery
	Solo laparoscopic surgery
	Resection of other organs
	invasion other organ
	adhesion
	Ascites
	EIPL
	total omentectomy
	Peritoneal lavage cytology
	celiac branch of vagus nerve preservation
	hepatic branch of vagus nerve preservation
	aberrant artery
	study
	Lymph nodedissection
Pathologic Report	No. of lesions
	Pathology report number
	WHO classifications
	WHO classifications detail
	AGC classifications
	EGC classifications
	Lauren's classifications
	Ming's classifications

	Lymphatic invasion
	Vascular invasion
	Perineural invasions
	Circular locations
	Tubular locations
	distance between cancer center and GE junction (if less than 5cm)
	Size (cm)
	Margin(p)
	Margin(d)
	stage detail
	T stage
	N stage
	M stage
	TNM stage
	No. of retrieved LNs
	No. of positive LNs
Postoperative information	IP chemotherapy
	SFD start
	hole n

Supplementary Table S4. Result of in-depth interview

Domain	Job type		
	Nurses	Administrators	Developers
**Prior experience and background knowledge**	I think RPA helps me with repetitive procedures in the beginning so that I can complete the task in about 30 minutes to	I got to know RPA for the first time when I oversaw the project in our team. I used to oversee computer security, and I	I think it's aptly called RPA because it allows more processes to perform their

	an hour. The task used to take three hours. I also had to open up all the charts. I need to open the chart, check the numbers, and make a table. I think that's the role of RPA that helps, which automates simple repetitive tasks.	thought that RPA was similar to the crawling system that scratches data, but I found out for the first time that it uses different technologies.	roles at the same time for simple tasks that humans had regularly processed.<br>I think there should be an order to standardize work through RPA, and I think I would explain that RPA is a concept that can help within the limits of operations where processes are established in a specific order.
**Context (Environment)**	Whatever it is, you can use it to do simple, repetitive tasks, for example, formatting the cancer registry that we're doing now in the medical records part. I think it's good that we can bring all the forms and test results and bring them up automatically by providing interlocking services and find and write what we have to use with our eyes. I think RPA automatically does what we had to do while charting our work.	I must extract the keywords that I want and enter them in the arm registry. That process is very difficult. Real doctors have a huge amount of knowledge. We need to code what they have and put it in RPA. So, it's very difficult. However, after the adoption of the RPA, I think it's better because I don't have to write such codes myself.	Later on, even if it's not something as big as cancer registration, people who were in real business administration positions think it's too messy, but there will be cases where they must do repetitive things.<br><br>I think the project to apply RPA to cancer registry purification is very good. If you can extract something from a specific text according to the rules, you can make it easier for the field to report. That's the advantage of RPA. It's relief from the simple and repetitive.
**Context (Attitude toward RPA)**	If RPA becomes smarter over time, it will be able to expand a little more than now, but I think we are still at a learning phase with the RPA at this stage. It's not that I don't trust RPA, but in order for it	I think it would be good to apply the RPA to tasks that are repetitive and do not require judgment in human decision making. To put it simply, during work, there are tasks that we must think a lot	About the process, I think RPA needs to be able to find the location of the size that I mentioned earlier, or find the letters of an icon, or if the screen suddenly gets smaller, then find the same

	to be smart, I have to pour the knowledge that we humans have into it. There are areas where this is easy and not so easy	about, so we have to make decisions, while there are tasks that we just do without thinking. I think there will be a lot of other tasks besides mine.	icon shape. I think you need this flexibility. If it can do all this, I think RPA is a good bot.
**Facilitating factors**	In fact, when you do it through the linked service, not this RPA, the values are not linked. When you press "Bring it in" and the value comes out empty, or if you see something strange, there are some parts where these corrections were actually in front of you, so there are times when the linkage is broken.	Other than "Cancer registry", I think it's a structure that was implemented in a hurry to see if you could capture a specific screen or not. In order to do this right, I think it would be necessary to call in a real web server that is able to check if the web service is running properly, give a true/false return value, or check the formal linkages, and other stuff like that. If that can happen, the system error analysis of the groupware login that we are currently having to do for the RPA can be made clearer. My opinion is that there is no clarity at the moment.	If the system screen changes frequently, maintenance of the RPA bot will also be a problem. RPA recognizes shapes like humans, but it can vary depending on the situation. The bot can find the red button after relocation, but sometimes the button cannot be found even if the color or text changes.
**Suggestion**	However, you don't have to disperse it to all departments, but I think one improvement that can be made is for the core members to brainstorm and install the RPA in the right department and the right place and continue to check thoroughly if it works.	When designing a new bot, we are trying to increase the probability that the RPA can detect abnormalities by referencing all previous experiences as much as possible through historical analysis.	I think the RPA would be more effective in finding the incomplete medical records than for other tasks.
